# The Home

**Published:** 1888-04

**Authors:** 


### THE HOME.

If called upon to give the most significant monosyllable in our language, it is not doubted but that by unanimous voice it would be Home. Its mention invariably pictures in the mind the old homestead, be it a cottage in a village street, or a mansion in the midst of ample grounds and costly environments.

Who does not revere the home of his childhood, and all the pleasant things that clustered about the family roof "in the days of auld lang syne," even to the pictures on the wall, the old arm-chair and the great overgrown family bible that rested with folded covers in its allotted place. There too was the little flower garden, with its neatly kept beds of merrigolds, pinks, pansies and bachelor buttons, with here and there a flowering rose bush that defied the winter snows. We seem to see them now, as we saw them years ago, and to realize more than ever that we shall never cease to love the picture, for it steals upon the memory with the freshness of spring days that renew the verdure of the fields and fill the world with rejoicing.

"Be it ever so humble there's no place like home"—no place that in the same degree rewards the care that is bestowed upon it, for the home is the nursery of the noble and great among the races of men. We cannot do too much for our homes. Every well chosen ornament within doors, and every shrub or flower that lends its beauty to the garden or the lawn, is an educator and sheds upon the whole household a refining influence which is felt in all the years to come. Who has not read "Picciola," and shared with the unfortunate captive his solicitude, for the companionship of the humble little plant that pushed its way above the rocky pavement of his prison yard, and who has not felt at some period of his life a like interest in some frail charge committed to his care and upon whose well being some dear hope was centered?

We have known parsimonious men in estimating the cost of living to reckon the interest upon the money value of the family homestead, as if this sacred spot was capable of being figured out in dollars and cents. As well might a sum be set upon a wife's devotion or the love of children. There has been erected in the park of a neighboring city a statue of Howard Paine. Other statues have been erected there in honor of men of greater renown, but of none whose single achievement has found such affectionate rooting in the bosoms of the young. To the little ones that gather about it, all that need be told them is that this is the image of the man who wrote the song of "Home, Sweet Home."

It is because the home is always sacred, and forms an ineffaceable picture in the mind, that we urge its sanctification by every available means. Nothing which adds to its comfort in a material sense, or to its adornment in a purely æsthetic relation, should be neglected. A single inexpensive but graceful picture in the living room, a stand of hardy flowers in a window are not without their educational as well as inspirational value. Such too are the harbingers of health, the warders of disease and oftentimes the forerunners of refinements which spiritualize the humblest lives and open the way to higher levels of thought and action.

There is much truth in the homely aphorism, "All work and no play makes Jack a dull boy." Children should be provided with the means of amusing themselves in suitable ways, and almost any innocent way is suitable. You cannot begin this too early, nor continue it too late. Playing cards are often objected to as implements of gambling, but so are wheat and flour and horses, and even men in tests of endurance sometimes coarse and brutalizing. But aside from cards there are various indoor amusements to which no reasonable objection can be made. By all means encourage seasonable outdoor exercises, and do not be alarmed at a little boisterousness. A hearty ringing laugh indicates a healthy condition. You will never hear it from the pale and sickly offspring of Dame Propriety who stands ready to check the least naturalness in her little old men and women. There is indeed nothing on earth so near heaven as a little child who has been suffered to retain its simplicity and naturalness. Pet animals are a great delight to the young. The care bestowed upon them is never without its reward, as the oft told tales of their fidelity and instinct that seems akin to reason, amply testify. Only the vicious or naturally cruel would indulge in their abuse, and to such, their companionship should be denied.

In a word, to the inmates of whatever age or station, the home should be made the dearest, sweetest place on earth. It is indeed the shield of honor and the sanctuary of love. Think not that anything which adds to its enjoyment is wasted. No investment yields such precious returns, since neither the love and gratitude of the young, nor the peace and contentment of the old, are capable of being estimated by the sordid values that obtain in the market places.

"How beautiful is Home ! The wanderer sees,
Returning from afar, the village spire,
And the ancestral roof, whose aged trees
Shelter perchance, wife, mother, child and sire,
Not their's the glory to which fools aspire.
The empty bauble, vainly called Renown ;
They are content to light the evening fire,
To feast on simple cheer, and lay them down
In joyous rest, to dream, unfearing fortune's frown."

